# News and Coming Events

**Published:** 1908-05-02

**Authors:** 


### NEWS AND COMING EVENTS.

The Royal Dental Hospital, Leicester Square, has received a donation of fifty guineas from his Majesty the King.

The Lord Mayor will preside at the annual festival dinner in aid of the German Hospital on Friday, May 22, at the Hôtel Metropole, London.

The number of names in the official Medical Register for this year is 39,827, being 298 more than at the end of 1906. The number of names in the corresponding issue of the "Medical Directory" (Churchill) is 39,703, which is 338 more than in the preceding issue.

The death is announced of Dr. Terrier, Professor of Clinical Surgery in the Paris Faculty of Medicine, and Surgeon to the Pitié Hospital. Professor Terrier's name is well known to English surgeons as that of a pioneer of aseptic surgery and an authority on the surgery of the biliary system.

Among the decorations conferred by the German Emperor on the occasion of his visit to England last November, and recently announced in the *Reichsanzeiger*, is the Order of the Crown, second class, granted to Sir Felix Semon, K.C.V.O., M.D., Physician Extraordinary to King Edward.

On April 27 Mr. Nathan Straus commenced a series of public demonstrations of his system of milk Pasteurisation at 67 Berners Street, Oxford Street, W. Mr. Straus has made considerable use of this method in the United States of America during the past 15 years as a philanthropic measure for the benefit of the poor. The demonstrations will continue each day from 2 to 5 p.m. until May 9.

The National League for Physical Education and Improvement has removed its offices to 11 Southampton Row, W.C., where it has entered into a working alliance with the British Institute of Social Service. In connection with the latter institution we are requested to announce that the number of inquiries dealt with last year was 980, and not 472, as stated on page 78 of our issue of April 18.

Under the terms of the will of the late Clifton Sorby, LL.D., F.R.S., of Sheffield, the University of Sheffield receives £10.000 to found a Professorship of Geology, while the Royal Society of London receives £5,000, the income from which is to be devoted to the establishment of a Fellowship for conducting original research, the object being the discovery of new facts rather than the teaching of what is known.

Dr. Charles Edward Underhill, President of the Royal College of Physicians, Edinburgh, died at Midlothian, on April 24, at the age of 63. Son of Mr. W. Lees Underhill, F.R.C.S., Tipton, Staffordshire, he was educated at Shrewsbury School, Caius College, Cambridge, and Edinburgh. Dr. Underhill was formerly Physician to the Royal Maternity Hospital, Edinburgh; Consulting Physician to the Royal Hospital for Sick Children; and President of the Royal Medical Society, the Obstetrical Society, and the Harveian Society, Edinburgh.

The King has been pleased to give directions for the appointment of J. E. Godfrey, Esq., M.B., Surgeon-General, and Chairman of the Local Government Board of British Guiana, to be a member of the Executive Council of that Colony.

Four lectures will be delivered at Gresham College, Basinghall Street, E.C., on Tuesday, May 5, Wednesday, May 6, Thursday, May 7, and Friday, May 8, by Dr. F. M. Sandwith, Gresham Professor of Physic. The first three lectures will be devoted to "The Prevention of Consumption"; the last lecture to "The Treatment of Consumption." The lectures are free to the public, and will begin each evening at six o'clock.

The medical members of the Departmental Committee appointed by the Home Secretary to inquire into the operation of the law relating to inebriates, and to their detention in reformatories and retreats, are as follows:—Dr. R. W. Branthwaite, Dr. H. E. Bruce Porter, Dr. H. B. Donkin, and Dr. C. A. Mercier. After this Committee, which is under the chairmanship of Sir J. Dickson-Poynder, Bart., M.P., have completed this inquiry, they will be reappointed, with certain changes, to investigate the value of existing methods of treatment of inebriety by the use of drugs.

The Lord Mayor of London presided on April 28 at the 101st annual festival of the City of London Truss Society at De Keyser's Hotel. He appealed to the British public to support this ancient charity, which has been in existence for more than 300 years. The Society, he added, did an enormous amount of good among the working classes and rendered immediate help. Some of the instruments supplied cost £5, and the cases came from all parts of the United Kingdom. The Society owed its bankers £2,500, and was greatly in need of more annual subscribers.

The suggestion recently made by Dr. Waldo, the Coroner for the City of London, that a refrigerator should be provided for London, wherein unclaimed bodies might be kept for the purpose of subsequent identification, is one that is worthy of consideration. Paris already possesses a public refrigerator for this purpose, which has been found of practical utility. We understand that this suggestion is now under consideration by the Court of Common Council. It is worthy of note that the London County Council has power, under section 93 of the Public Health (London) Act of 1901, to provide and fit up one or two suitable buildings, to which dead bodies found in London and not identified may, on the order of a coroner, be removed and preserved for ultimate identification.

Lord Ludlow, Treasurer of St. Bartholomew's Hospital, in his report for 1907, states that the new Out-Patients' Block opened by the Prince of Wales in July last is now in full working order, and has fully realised all expectations as to the efficiency of the accommodation it provides. Another important step in the scheme of reconstruction entered upon during the past year was the commencement of the Pathological Block. The Treasurer's report adds that the receipts, both for the Out-Patients' Block and for the Pathological Block, have been exhausted, and that advances have had to be obtained to meet the payments to contractors. A sum of not less than £35,000 is still needed to repay these advances and to provide the balance due to contractors on account of the Out-Patients' Block and to defray the cost of erection of the Pathological Block.

In the preliminary drafts of the charters for the new Dublin and Belfast Universities, under the provisions of the Irish Universities Bill, the following members of the medical profession appear upon the schedules of names for the first Senates :—For the University to have its seat in Dublin : D. J. Coffey, M.A., M.B., B.Ch. (President of the Constituent College in Dublin), M. F. Cox, M.D., A. Dempsey, M.D., J. S. M'Ardle, M.Ch., Sir C. Nixon, Bart, M.D., LL.D., J. F. O'Carroll, M.D., P. T. O'Sullivan, M.D., B.Ch., C. Yelverton Pearson, M.D., M.Ch., J. P. Pye, M.D., G. Sigerson, M.D., B. C. A. Windle, M.A., M.D., D.Sc., F.R.S. (President of the Constituent College in Cork). For the University, to have its seat in Belfast : Johnson Symington, M.D., F.R.S., F.R.S.E. (Registrar), Peter R. O'Connell, M.D. (High Sheriff of Belfast), John Park, M.D., Litt.D., Sir William Whitla, M.A., M.D., LL.D., Thomas Sinclair, M.D., M.Ch., F.R.C.S.Eng., John Walter Browne, B.A., M.D., and Peter Redfern, M.D., F.R.C.S.Eng.

The Incorporated Institute of Hygiene commenced on April 27 a special exhibition of a "model public-house" at 34 Devonshire Street, Harley Street, which will continue on view until May 9. The object of the Institute is the demonstration of how brighter and better surroundings might conduce to sobriety. The exhibition is modelled on the lines of the Continental café, where a higher tone, more hygienic conditions, and greater moderation generally prevail than in the lower class of public-house in this country. The prominent feature is that there is no drinking at the bar, non-alcoholic beverages are served as freely as alcoholic refreshments, and particular attention is given to the serving of food, and to the provision of literature and games. Sir William Bennett, in declaring the exhibition open, said that if the Government, when considering the modification of the present licensing system, would formulate some scheme for the bettering of the environment of the drinker, especially in the lower type of public-house, more would be done towards the promotion of temperance than any amount of restrictive legislation, however drastic. Dr. Clement Godson, Dr. Murray Leslie, Dr. Thresh, and Mr. Mayo Robson also spoke in support of this attempt to improve the public-house.

### Medical Motorists.

The Motor Union has appointed from among its members the following committee of medical motorists to protect the interests of the general body of medical men using motor-cars and to advise the Motor Union on all matters in which the interests of medical men as motorists are particularly concerned : Mr. C. B. Lockwood, F.R.C.S. (London), Mr. W. Watson Cheyne, C.B. (London), Dr. C. Barker (Watford), Dr. Lovell Drage (Hatfield), Dr. E. L. Rowe (Ipswich), Dr. Moss-Blundell (Thornton Heath), Dr. Russell Matthews (Peckham), Mr. J. R. Benson, F.R.C.S. (Bath), Dr. W. G. Gilpin (Bourne), Dr. W. F. Burgess (Streatham), Mr. L. A. Bidwell, F.R.C.S. (London), Dr. J. Lewis Lock, M.A. (Uxbridge), Mr. E. W. Marshall, M.R.C.S. (Mitcham), Mr. W. E. C. Musson, F.R.C.S. (Hammersmith), Dr. J. Hopkins Walters (Reading), Dr. P. P. Whitcombe (London), Dr. C. Buttar (London), Dr. H. Myddelton-Gavey (Eastbourne), Dr. O. R. M. Wood (Woolpit), Dr. Holroyd (Pannal), Dr. A. T. Falwasser (Maidstone), Dr. Travers (Maidstone), Dr. G. Horatio Jones (Deddington), Dr. A. Lindow, M.R.C.S. (Plumstead), Dr. Priestly Leech (Halifax), Dr. D. J. Mason (Maidenhead), and Dr. A. Page-Robertson (Glasgow). At a meeting held at the offices of the Union, 1 Albemarle Street, Piccadilly, W., Mr. C. B. Lockwood, F.R.C.S., was unanimously elected Chairman of the committee, and Dr. J. L. Lock, M.A., M.B., Vice-Chairman. The question of the position of doctors in reference to the proposed taxation of motor-cars was considered, and it was resolved that a letter be sent to the Chancellor of the Exchequer signed by all members of the committee, embodying the following resolution : "That in the opinion of medical motorists any additional taxation upon motor-cars would not be equitable, and would press with special severity upon medical men who use motor vehicles to enable them to carry out their professional duties." The new charges of the Metropolitan Water Board for water for washing cars was considered, and it was resolved that the proposed charge of 20s. per car per annum was excessive and unfair as compared with the rate for water for horse carriages, and it was resolved to approach the Board upon the matter.

